# Targeting of the Nasal Mucosa by Japanese Encephalitis Virus for Non-Vector-Borne Transmission

**DOI:** 10.1128/JVI.01091-18

**Published:** 2018-11-27

**Authors:** Obdulio García-Nicolás, Roman O. Braun, Panagiota Milona, Marta Lewandowska, Ronald Dijkman, Marco P. Alves, Artur Summerfield

**Affiliations:** a Institute of Virology and Immunology, Mittelhäusern, Switzerland; b Graduate School for Cellular and Biomedical Sciences, University of Bern, Bern, Switzerland; c Department of Infectious Diseases and Pathobiology, Vetsuisse Faculty, University of Bern, Bern, Switzerland; University of Texas Southwestern Medical Center

**Keywords:** Japanese encephalitis virus, direct contact transmission, macrophages, nasal epithelial cells, pig

## Abstract

The mosquito-borne Japanese encephalitis virus (JEV) causes severe central nervous system diseases and cycles between *Culex* mosquitoes and different vertebrates. For JEV and some other flaviviruses, oronasal transmission is described, but the mode of infection is unknown. Using nasal mucosal tissue explants and primary porcine nasal epithelial cells (NEC) at the air-liquid interface (ALI) and macrophages as *ex vivo* and *in vitro* models, we determined that the nasal epithelium could represent the route of entry and exit for JEV in pigs. Porcine NEC at the ALI exposed to with JEV resulted in apical and basolateral virus shedding and release of monocyte recruiting chemokines, indicating infection and replication in macrophages. Moreover, macrophages stimulated by alarmins, including interleukin-25, interleukin-33, and thymic stromal lymphopoietin, were more permissive to the JEV infection. Altogether, our data are important to understand the mechanism of non-vector-borne direct transmission of Japanese encephalitis virus in pigs.

**IMPORTANCE** JEV, a main cause of severe viral encephalitis in humans, has a complex ecology composed of a mosquito-waterbird cycle and a cycle involving pigs, which amplifies virus transmission to mosquitoes, leading to increased human cases. JEV can be transmitted between pigs by contact in the absence of arthropod vectors. Moreover, virus or viral RNA is found in oronasal secretions and the nasal epithelium. Using nasal mucosa tissue explants and three-dimensional porcine nasal epithelial cells cultures and macrophages as *ex vivo* and *in vitro* models, we determined that the nasal epithelium could be a route of entry as well as exit for the virus. Infection of nasal epithelial cells resulted in apical and basolateral virus shedding and release of monocyte recruiting chemokines and therefore infection and replication in macrophages, which is favored by epithelial-cell-derived cytokines. The results are relevant to understand the mechanism of non-vector-borne direct transmission of JEV.

## Abstract (precis)

JEV, a main cause of severe viral encephalitis in humans, has a complex ecology composed of a mosquito-waterbird cycle and a cycle involving pigs, which amplifies virus transmission to mosquitoes, leading to increased human cases. JEV can be transmitted between pigs by contact in the absence of arthropod vectors. Moreover, virus or viral RNA is found in oronasal secretions and the nasal epithelium. Using nasal mucosa tissue explants and three-dimensional porcine nasal epithelial cells cultures and macrophages as *ex vivo* and *in vitro* models, we determined that the nasal epithelium could be a route of entry as well as exit for the virus. Infection of nasal epithelial cells resulted in apical and basolateral virus shedding and release of monocyte recruiting chemokines and therefore infection and replication in macrophages, which is favored by epithelial-cell-derived cytokines. The results are relevant to understand the mechanism of non-vector-borne direct transmission of JEV.

## INTRODUCTION

Japanese encephalitis virus (JEV) is responsible for the most important viral encephalitis affecting humans, with a yearly estimation of 68,000 cases and with a mortality rate of 14% to 21% (1). Importantly, about 30% to 50% of the surviving encephalitis patients can suffer severe long-term neurological sequelae (2). JEV is a positive single-stranded RNA virus belonging to the genus *Flavivirus* and is currently endemic in the rice-growing areas of North, Southeast, and South Asia. JEV is predominantly maintained kept in an enzootic cycle between mosquitoes of the genus *Culex* and vertebrate hosts, in particular certain waterbirds and pigs as maintenance or amplifying hosts (2–5).

Pigs are highly susceptible to JEV infection and rapidly develop viremia, which can last for a few days, during which they can transmit the virus to mosquitoes (4, 6). Infected pigs normally present mild neurological symptoms, but the infection of pregnant sows can cause abortions and stillbirth (7). Although JEV infection is typically mosquito borne, contact transmission and high susceptibility to oronasal infection have been described (8). Several *in vivo* experiments demonstrated that JEV can be excreted oronasally in infected pigs, independently of the route of inoculation, for a period of 5 days, with a maximum level of virus shedding reached after the end of viremia (8–12). Furthermore, mathematical modeling using data from outbreaks in Cambodian pig farms supported the possibility that direct transmission also occurs under field conditions (13). These findings represent a warning that JEV could have the potential to continue to circulate in the pig population by direct transmission in temperate regions during cold mosquito-free periods. In fact, a reemergence of JEV in pig farms located in temperate areas after the winter season without the presence of vectors has been reported in Hokkaido Island in northern Japan (14). It is also important to note that vector-free transmission of mosquito-borne flaviviruses is not restricted to JEV and pigs. Several species, including macaques, mice, hamsters, guinea pigs, rats, and squirrel hamsters, are susceptible to oronasal infection with JEV (15–17). In addition, non-vector-borne transmission has been described for other flaviviruses (18, 19). Examples are Zika virus (ZIKV) (20, 21), West Nile virus (WNV) (22, 23), St. Louis encephalitis virus (SLEV) (24), Bagaza virus (BAGV) (25), Tembusu virus (TMUV) (26), and Wesselsbron virus (WESSV) (27).

Considering these observations, the present study addressed the potential role of the nasal epithelium in contact transmission. Our hypothesis was based on the observation that after oronasal JEV challenge of immune pigs, it is possible to detect the challenge virus in oronasal swabs for several days in the absence of detectable viremia indicating local virus replication (9). While JEV is also found in tonsils (8, 9, 12), immune animals’ tonsils were found to be protected from infection, indicating that this organ is infected only following viremia (9) and would therefore not represent the point of JEV entry. On the other hand, a recent study demonstrated the presence of viral RNA in the nasal epithelium as well as in the olfactory neuroepithelium (11). We therefore investigated whether the nasal epithelium can be infected from the apical surface and whether this infection would cause both apical and basolateral virus shedding. This would indeed represent a prerequisite for the epithelium of the upper respiratory tract to play a role in direct transmission of JEV. The present work included *ex vivo* and *in vitro* experimental models as porcine nasal explants and well-differentiated porcine primary nasal epithelial cells (NEC) cultured at the air-liquid interface (ALI). In fact, our data demonstrate that JEV has the ability of infecting apically, resulting in both apical and basolateral virus shedding in swine nasal epithelial cells and indicating that the porcine nasal mucosa could represent a gateway for JEV entry and exit in pigs. Furthermore, we demonstrate that epithelial cells release soluble factors favoring subsequent infections of macrophages known to be present in the lamina propria of the mucosa.

## RESULTS

### JEV replicates in porcine nasal mucosa explants.

**FIG 1:**
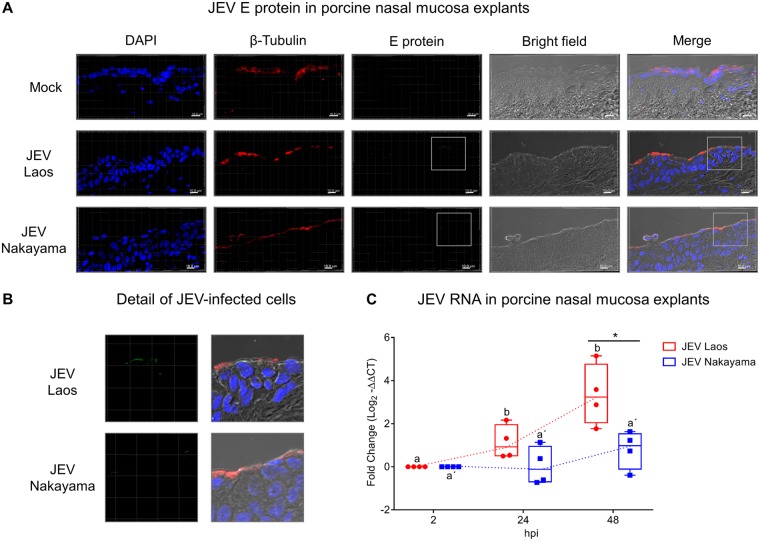
JEV replicates in porcine nasal explants. Porcine nasal mucosa explants were challenged with JEV Laos or Nakayama at 10^5^ TCID_50_/sample for 2 h, followed by wash steps and culture in medium for 48 h. In panel A, nuclei were stained with DAPI (blue), cilia with anti-β-tubulin (red), and JEV with anti-E protein 4G2 (green). The scale bar represents 10 µm. Three-dimensional (3D) scans were acquired using confocal microscopy. In panel B, a higher magnification of panel A is shown. In panel C, viral RNA was quantified by real-time RT-PCR. Results were calculated as fold change of the threshold cycle and are represented as 25 to 75% interquartile boxes showing the mean and 95% confidence intervals. Different superscript letters indicate significant difference (*P* ≤ 0.05) between samples challenged with the same JEV strain (letters without or with apostrophe for JEV Laos and Nakayama strain, respectively); significant differences between distinct JEV strains at the same time postinfection are shown by an asterisk.

As a first step, we challenged porcine nasal mucosa explants from four different pig donors with both JEV genotype 1 strain Laos and genotype 3 strain Nakayama, in comparison to mock control (Fig. 1). After 48 h postinfection (hpi), JEV envelope (E) protein was detected in epithelial cells (Fig. 1A and B), with a more efficient infection found with the Laos strain-challenged tissues. An increase in JEV RNA loads from both strains was also detected in porcine nasal mucosa, indicative of virus replication (Fig. 1C). JEV Laos RNA showed an increase in expression from 24 hpi on, further increasing at 48 hpi. The Nakayama strain displayed a slower viral RNA increase, with only three out of four positive samples 48 hpi (Fig. 1C).

### JEV efficiently infects primary porcine NEC.

**FIG 2:**
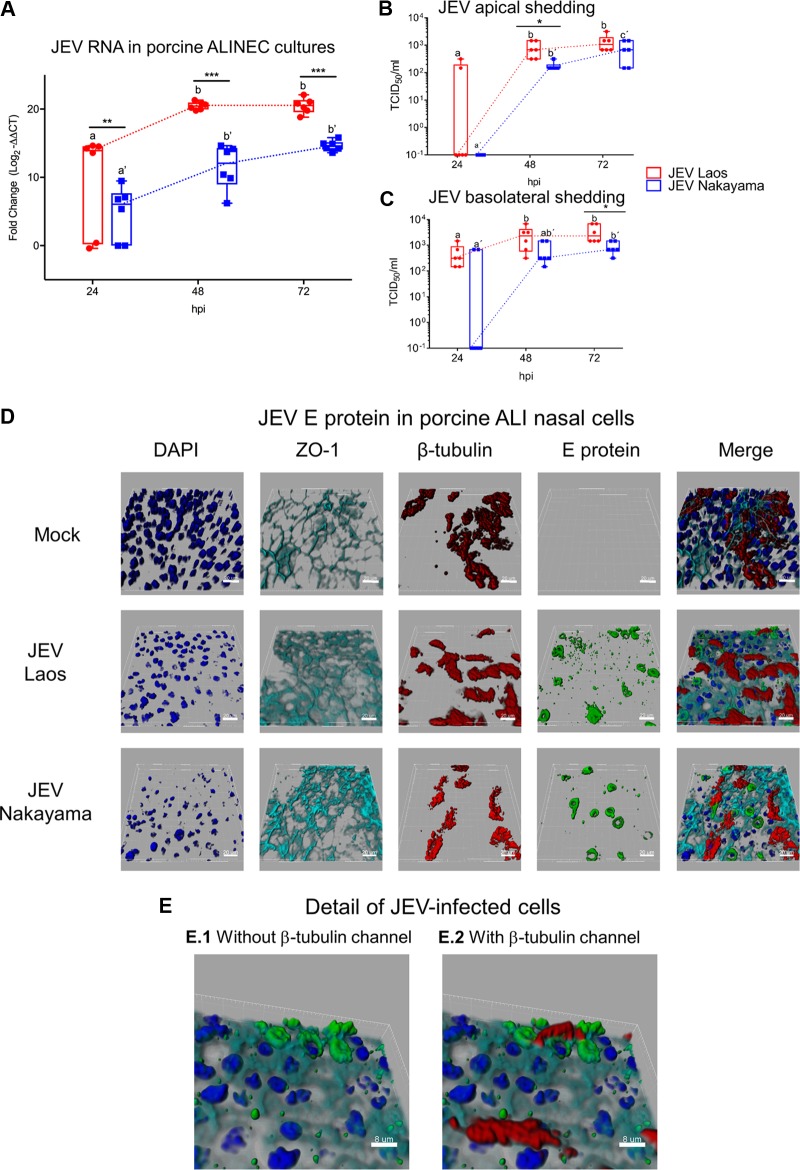
JEV infects and replicates in porcine NEC. In panel A, porcine NEC cultures were infected with JEV strains at MOI of 0.1 TCID_50_/cell, and after 24, 48, and 72 hpi, inserts were collected and viral RNA was quantified by real-time RT-PCR. In panels B and C, viral titers from the apical and basolateral compartments of the same cultures as in panel A are shown. In panels D and E, JEV-infected porcine NEC were analyzed by multicolor immunostaining for nuclei (DAPI, dark blue), cilia (β-tubulin, red), tight junctions (ZO-1; light blue), and JEV E protein. 3D scans were acquired using confocal microscopy; scale bars represent 20 and 8 µm for panels D and E, respectively. The experiment was repeated three times in duplicate. In panels A to C, results are represented as 25 to 75% interquartile boxes showing the means and 95% confidence intervals. Different superscript letters indicate significant differences (*P* < 0.05) between samples challenged with the same JEV strain (letters without and with apostrophe for the Laos and Nakayama strains, respectively). Significant differences between distinct JEV strains at the same time postinfection are shown by an asterisk for virus titers (A) and for RNA levels (B and C).

To confirm and further investigate the ability of JEV to infect porcine NEC from their apical side, we utilized well-differentiated ALI cultures established from porcine nasal mucosa. These experiments showed that both JEV strains infected cultures from the apical side, resulting in virus replication (Fig. 2). Again, the Laos strain showed statistically significantly higher levels of viral RNA than the Nakayama strain. Despite that, the two JEV strains tested displayed similar replication profiles, reaching a maximum at 72 hpi (Fig. 2A). To test for apical and/or basolateral live virus shedding upon infection, we collected basolateral chamber culture media and performed apical washings of the infected NEC. The results demonstrate that JEV was apically shed after infection with both JEV strains (Fig. 2B). Interestingly, similar JEV titers were detected in the culture media collected from the basolateral and apical sides of the inserts (Fig. 2C), supporting the idea that infection of epithelial cells could result in a systemic infection. With respect to RNA levels, the maximum titers were reached at 72 hpi in both compartments of the ALI system. At 72 hpi, as shown in Fig. 2D, we visualized JEV infection of NEC by labeling of virus (E protein, green), nuclei (4′,6-diamidino-2-phenylindole dihydrochloride [DAPI], dark blue), tight junctions (ZO-1, light blue), and cilia (β-tubulin, red). While we observed mainly isolated E protein-positive cells for the Nakayama strain, the Laos strain led to the formation of clusters of E protein-positive cells (Fig. 2D) and colocalization of E protein and β-tubulin signals, suggesting replication in ciliated epithelial cells (Fig. 2E).

### JEV infection of NEC is associated with limited cell death.

**FIG 3:**
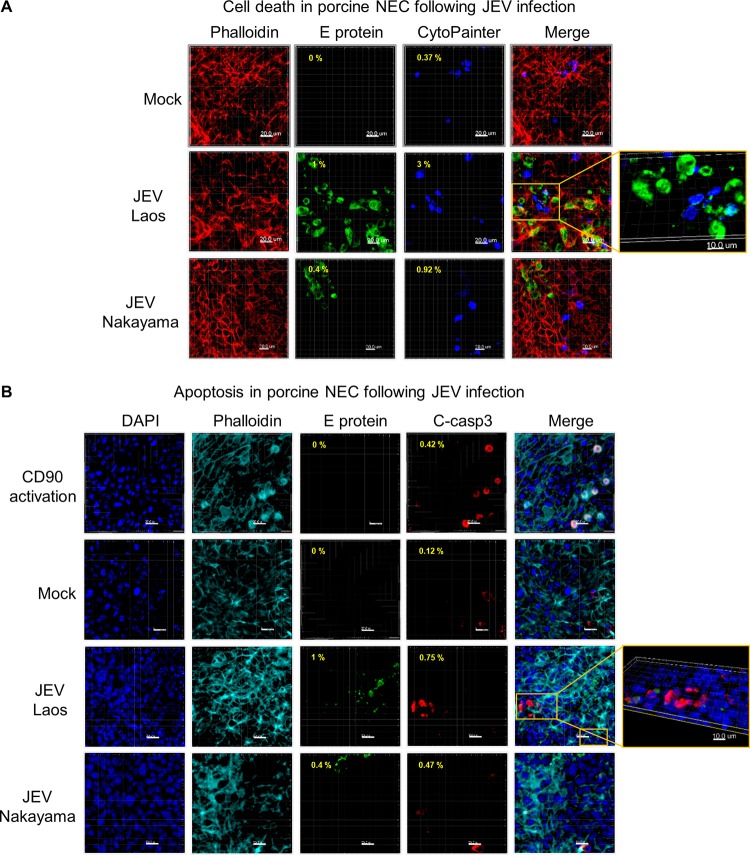
JEV-induced cell death in porcine NEC. Porcine NEC were infected with JEV Laos and Nakayama at an MOI of 0.1 TCID_50_/cell and incubated for 72h. In panel A, dead porcine NEC were labeled with CytoPainter (dark blue) before fixation and then cells were immunolabeled for actin (red) and for JEV E protein (green). In panel B, we immunolabeled cleaved caspase 3 (red), actin (light blue), JEV E protein (green), and nuclei (DAPI, dark blue). CD90-induced apoptosis induction was employed as a positive control. The percentage of E protein expressing dead (A) or apoptotic (B) cells are shown in the upper left corner of each image. 3D scans were acquired using confocal microscopy. The scale bar represents 20 µm. Detailed images of necrotic and apoptotic cells are shown to the far right (A and B).

In NEC cultures, JEV induced limited cell death, visualized as increased membrane permeability (dark blue in Fig. 3A). This cell death was at least partially mediated by apoptosis since cleaved caspase 3 was detected in the infected cultures (red in Fig. 3B). Relating to the higher rate of infection, the Laos strain induced more cell death than the Nakayama strain.

### JEV induces poor innate immune responses in NEC.

**FIG 4:**
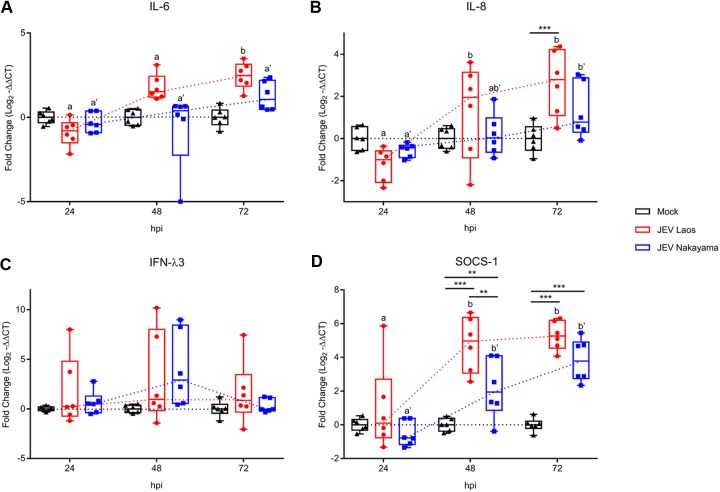
JEV induces weak proinflammatory responses following infection of porcine NEC. NEC were challenged with JEV Laos and Nakayama at an MOI of 0.1 TCID_50_/cell. After 24, 48, and 72 hpi, inserts were collected and cytokine gene expression was quantified by RT-qPCR for IL-6 (A), IL-8 (B), IFN-λ3 (C), and SOCS1 (D). The experiment was repeated three times in duplicate. Data are shown as 25% to 75% interquartile boxes with means and 95% confidence intervals. Different superscript letters indicate significant difference (*P* < 0.05) between samples challenged with the same JEV strain (letters without and with apostrophe for JEV Laos and JEV Nakayama, respectively); differences between distinct JEV strains and the mock control at the same time postinfection are shown by asterisks (*, *P* ≤ 0.05; **, *P* ≤ 0.002; ***, *P* ≤ 0.001).

**FIG 5:**
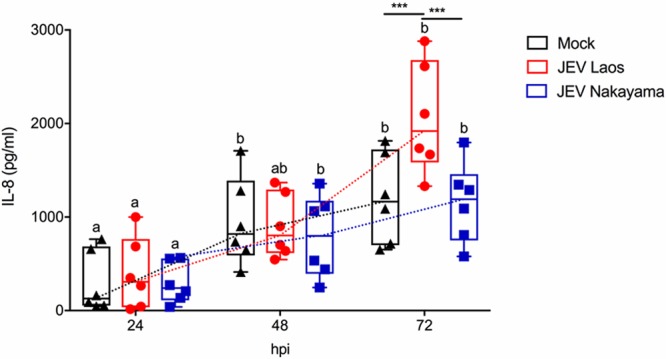
JEV Laos induces delayed IL-8 secretion following infection of porcine NEC. Porcine NEC were challenged with JEV Laos and Nakayama at an MOI of 0.1 TCID_50_/cell. After 24, 48, and 72 hpi, basolateral supernatants were collected and IL-8 levels were determined by ELISA. The experiment was repeated three times in duplicate. Data are shown as 25% to 75% interquartile boxes with means and 95% confidence intervals. Different superscript letters indicate significant difference (*P* < 0.05) between samples challenged with the same JEV strain (letters without and with apostrophe for JEV Laos and JEV Nakayama, respectively); differences between distinct JEV strains and the mock control at the same time postinfection are shown by asterisks (*, *P* ≤ 0.05; **, *P* ≤ 0.002; ***, *P* ≤ 0.001).

Following infection of airway epithelial cells by respiratory viruses, such as influenza virus, rhinovirus, parainfluenza virus type 1, respiratory syncytial virus (RSV), or severe acute respiratory syndrome (SARS) coronavirus, several proinflammatory cytokines are induced, including interleukin-1β (IL-1β), IL-6, IL-8, and interferon lambda 1 to 3 (IFN-λs) (28–35). We therefore assessed the innate immune response following JEV infection of NEC. Only JEV Laos induced significant IL-6 gene expression at a late time point (72 hpi [Fig. 4A]). Both JEV strains induced a significant induction of IL-8 mRNA at 48 and 72 hpi (Fig. 4B). Nevertheless, at the protein levels no virus-induced IL-1β, IL-6, or tumor necrosis factor (TNF) was detected at any of the time points tested (data not shown). Interestingly, IL-8 secretion was enhanced at 72 hpi (*P* ≤ 0.001) in JEV Laos-infected NEC (2,055 ± 595 pg/ml) compared with those in the mock control (1,201 ± 595 pg/ml) and JEV Nakayama (1,151 ± 595 pg/ml) challenges (values are means ± standard deviations [SD]) (Fig. 5).

Although IFN-λ3 mRNA was upregulated in a few samples, neither of the JEV strains induced a statistically significant increase in expression for this gene at the investigated time points, suggesting an impact of JEV infection on the IFN pathway response (Fig. 4C). It has been described that suppressor of cytokine signaling 1 (SOCS1), a negative regulator of IFN responses, is upregulated in cells infected by JEV (36). Interestingly, 48 to 72 hpi both JEV strains induced a significant upregulation of SOCS1 in NEC, with higher gene expression in Laos-infected cells (Fig. 4D).

### JEV infection of NEC induces chemokines mediating monocyte recruitment.

**FIG 6:**
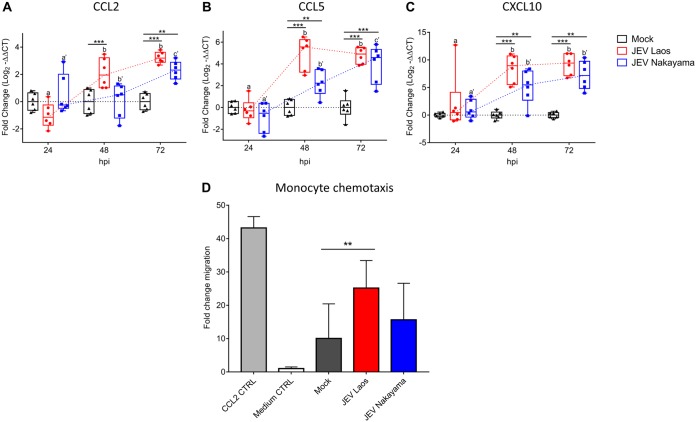
JEV-infected porcine NEC express chemokines and induce monocyte chemotaxis. RNA was extracted from NEC and gene expression of chemokines was determined by RT-qPCR for CCL2 (A), CCL5 (B), and CXCL10 (C). Data are shown as 25% to 75% interquartile boxes with means and ±95% confidence intervals. Basolateral medium from porcine NEC at 72 hpi was used for chemotaxis experiments. (D) Mean values ± SD are shown, with different superscript letters indicating significant difference (*P* ≤ 0.05) between samples challenged with the same JEV strain (letters without and with apostrophe for JEV Laos and JEV Nakayama, respectively); differences between distinct JEV strains and mock at the same time postinfection are shown by asterisks (*, *P* ≤ 0.05; **, *P* ≤ 0.002; ***, *P* ≤ 0.001).

Considering that the establishment of innate immune responses by virus-infected cells may also involve the production of chemokines responsible for the recruitment of immune cells to the site of infection, we measured the gene expression of several chemokines in JEV-infected porcine NEC (Fig. 6). mRNA expression levels of CCL2, CCL5, and CXCL10 were significantly upregulated by the virus at 48 and 72 hpi compared to those in mock controls (Fig. 6A to C). Nevertheless, for CCL2, only JEV Laos induced a significant mRNA upregulation at 48 hpi (Fig. 6A).

CCL2, CCL5, and CXCL10 have been described as important chemokines for monocyte recruitment (37–39). Consequently, we determined the monocyte chemoattractant activity of basolateral medium collected from JEV-infected NEC at 72 hpi (Fig. 6D). We observed that medium from Laos strain-infected NEC induced a significant increase of porcine monocyte migration (Fig. 6D).

### IL-4 and factors released from NEC such as alarmins enhance macrophages infection by JEV.

Considering that JEV was shed to the basolateral side of the NEC and that such cultures released monocyte chemoattractant factors, we next investigated infection of monocyte-derived macrophages (MDM) to mimic the possible subsequent *in vivo* cellular target following infection of NEC. Importantly, resting mucosal tissue resident macrophages have been described to have an “M2-like” functional status resembling that of IL-4-polarized macrophages (40, 41). We therefore tested the infectivity of JEV released from NEC cultures for both nonpolarized and IL-4-polarized macrophages. To also assess potential additional factors released from the infected NEC cultures, we compared the infectivity of basolateral culture media obtained from infected NEC and JEV derived from Vero cell-propagated virus. Of note, the media used as infectious JEV sources were adjusted to the same multiplicity of infection (MOI).

**FIG 7:**
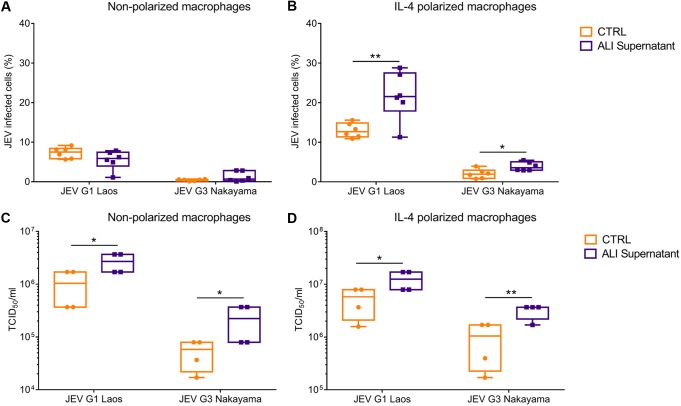
Porcine NEC cultures release factors enhancing porcine macrophage infection by JEV. Basolateral supernatants from NEC at 72 hpi were used to infect nonpolarized and IL-4-polarized macrophages. The percentages of infected cells are shown for nonpolarized (A) and IL-4-polarized macrophages (B). Panels C and D show the virus shed from nonpolarized and IL-4-polarized macrophages, respectively. Results represent those from three independent experiments performed in duplicate. Data are shown as 25% to 75% interquartile boxes with means and 95% confidence intervals. Different superscript letters indicate significant difference (*P* < 0.05) between samples challenged with the same JEV strain (letters without and with apostrophe for JEV Laos and JEV Nakayama, respectively); differences between distinct JEV strains and the mock control at the same time postinfection are shown by asterisks (*, *P* ≤ 0.05; **, *P* ≤ 0.002).

**FIG 8:**
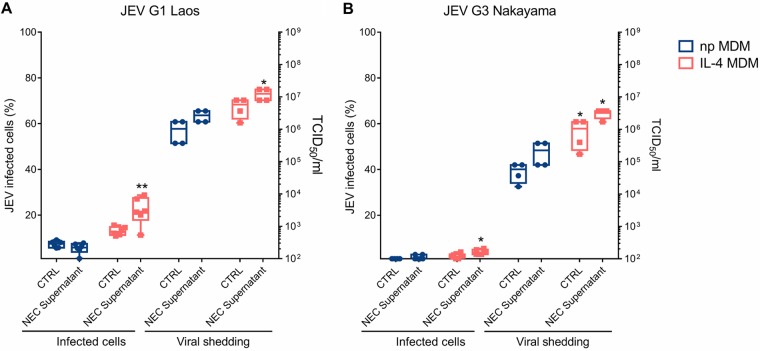
JEV infection is enhanced in IL-4-treated MDM. Porcine MDM were infected with JEV Laos obtained from virus stocks or from the basolateral medium of porcine NEC cultures infected with JEV Laos or Nakayama for 72 h at an MOI of 1 TCID_50_/cell. After 24 h, E expressing cells were determined by flow cytometry and virus titers in the supernatants measured for JEV Laos and Nakayama (A and B, respectively). All experiments were repeated three independent times in duplicate, and data are represented as 25% to 75% interquartile boxes showing the means and 95% confidence intervals. Significant differences between nonpolarized and IL-4 polarized MDM for the same challenge condition are shown by asterisks (*, *P* ≤ 0.05; **, *P* ≤ 0.002).

The first observation made in these experiments was that IL-4 treatment of macrophages enhanced infectivity and virus replication of both strains of JEV (Fig. 7A and Fig. 8). The second observation was that JEV harvested from NEC had enhanced infectivity for IL-4-treated macrophages (Fig. 7A) and resulted in higher levels of replication in all macrophage cultures than obtained with virus grown in Vero cells (Fig. 7B). This indicated the presence of additional factors released by epithelial cells with a potential to enhance infection of macrophages.

**FIG 9:**
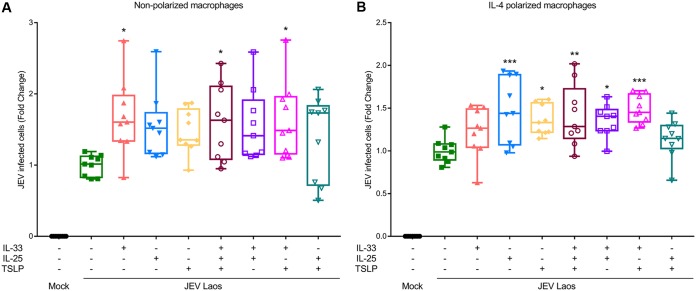
IL-25, IL-33, and TSLP can enhance infection of MDM. Nonpolarized (A) and IL-4-polarized (B) porcine macrophages were challenged with JEV Laos at an MOI of 1 TCID_50_/cell. The influence of IL-25, IL-33, and TSLP (all at 10 ng/ml) or combinations thereof was tested when added with the virus inoculum, incubated for 1.5 h, and washed off; after 24 hpi, cells were harvested and E protein-positive cells acquired by flow cytometry. Data represent those from three independent experiments carried out in triplicate and illustrate 25% to 75% interquartile boxes showing the means and 95% confidence intervals. Differences between JEV infection of cells in the presence or absence of the cytokines are shown by asterisks (*, *P* ≤ 0.05; **, *P* ≤ 0.002; ***, *P* ≤ 0.001).

Epithelial cells from the respiratory tract release innate cytokines in response to a viral infection, also known as “alarmins,” which include IL-33, IL-25, and thymic stromal lyphoprotein (TSLP); these cytokines activate immune cells like T cells, dendritic cells, and macrophages, normally driving a Th2 immune response (42–45). We therefore investigated the impact of these alarmins which could be potentially released by JEV-infected epithelial cells. To this end, we tested the effects of human recombinant IL-25, IL-33, and TSLP on macrophage infectivity by JEV. These experiments showed that the presence of the alarmins during the viral infection phase can lead to a higher percentage of infected cells (Fig. 8A). In nonpolarized macrophages, this enhancement of infection by JEV was statistically significant for IL-33 alone, for IL-33 combined with TSLP, and for IL-33 combined with TSLP and IL-25 (Fig. 9A). In IL-4-polarized macrophages, a further enhancement of infection was statistically significant for IL-25 alone, TSLP alone, and every combination of IL-33 with the other alarmins (Fig. 9B).

## DISCUSSION

As mentioned in the introduction, there is increasing evidence for vector-free transmission of flaviviruses, in which oronasal, contact, or aerosol exposure to the virus has resulted in infection of various vertebrates. The reported flaviviruses, all described as mosquito borne, include at least JEV, WNV, SLEV, yellow fever virus, ZIKV, Spondweni virus, WESSV, TMUV, and dengue virus (DENV), and the species concerned include pigs, humans, nonhuman primates, mice, guinea pigs, hamsters, geese, partridges, ducks, several other species of birds, and even alligators (8, 9, 12, 15–17, 20–22, 24–27, 46–54). Some of these reports are from experimental studies, and others are from field observations, laboratory infections, or infections of exposed health care providers or animal caretakers. Despite this multitude of reports, nothing is known about the mechanisms leading to such routes of transmission. For nonlaboratory exposures, a first prerequisite for aerosol contact transmission would be that the virus is secreted into body fluids such as mucosal secretions or urine. In fact, this has been described in many of the references previously cited, but the source of virus and first target remained unknown.

**FIG 10:**
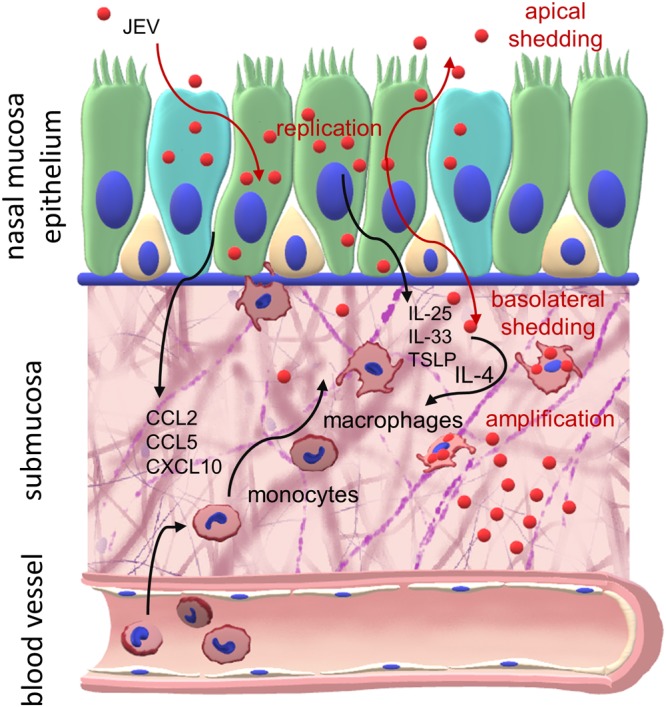
Targeting of the nasal mucosa by JEV. Shown is a graphical summary of the interaction of JEV with NEC and myeloid cells.

Considering the above, the present study addressed JEV infection of the porcine airway mucosal epithelium, which represents an important first physical barrier for the entrance of pathogens into hosts. In addition, epithelial cells also contribute to innate antiviral responses through the production of cytokines, chemokines, and IFNs (55–57). To also address mucociliary activities, we used well-differentiated primary NEC cultures and demonstrated that JEV infects epithelial cells from nasal mucosal explants as well as NEC cultures (summarized in Fig. 10). Despite the fact that respiratory viruses are normally shed only apically and rarely basolaterally, JEV is shed to both the apical and basolateral sides of the epithelial cells and such an infection could thus mediate virus entry into the host as well as oronasal virus spread to other hosts in a manner principally comparable to that of respiratory viruses such as influenza virus. Our *in vitro* results complements previously published *in vivo* findings, which can explain the high efficiency of JEV infection of pigs via the oronasal route and are in line with different experiments which showed oronasal shedding several days beyond the end of viremia, and they additionally can explain the detection of high levels of JEV RNA in nasal mucosa, which can last up to 10 days after challenge (8–12). The *in vivo* relevance of the presented data is further supported by the observation that after oronasal JEV challenge of immune pigs, it is possible to detect the challenge virus in oronasal swabs for several days in the absence of detectable viremia (9), pointing to a local cellular source for virus replication, which is also supported by a recent study in which the larger JEV RNA amount was detected in nasal mucosa (11).

We also investigated innate immune responses induced by JEV infection of porcine NEC, and our data indicate a mild and delayed induction of the proinflammatory cytokines IL-6 and IL-8 and undetectable levels of other cytokines, such as IL-1β and TNF, contrasting with the inflammatory cytokine responses induced by common respiratory viruses (58–60). As for IFN-λs, which are important mucosal antiviral factors produced by respiratory epithelial cells, we did not detect a significant increase at the transcriptional level, confirming the relatively silent infection of the nasal epithelium, which could enable JEV to bypass the first line of immune defenses of the upper respiratory tract. This idea is also supported by the weak cytopathic effect of JEV on NEC and by the observed induction of SOCS1, a potent negative regulator of the IFN pathway and consequently also of the expression of IFN-stimulated genes (61). This is in accordance with a report describing SOCS1 induction during JEV infection (36).

As part of the innate immune response against virus infections targeting the respiratory tract, such as influenza virus or RSV infection (28, 30, 32), JEV infection of porcine NEC also induced several chemokines (CCL2, CCL5, and CXCL10), which would direct the migration of monocytes to the site of infection. This led us to investigate the potential next step for the establishment of a systemic infection, which could be the infection of myeloid cells present in the submucosal area. In fact, MDM were found to be susceptible to infection and supported a high level of virus replication which was further enhanced by IL-4 polarization. This is relevant, as such a polarization could also be induced following JEV infection of NEC through the release of alarmins, such as IL-33, IL-25, and TSLP, from stressed and dying epithelial cells (42–45). These alarmins are known to stimulate immune cells present in the nasal mucosa to produce IL-4 and IL-13, which promote M2-like macrophage polarization (62–65). In fact, our data indicate that supernatants from infected porcine NEC cultures contained factors which further enhanced infection of macrophages. We also demonstrated that these alarmins can enhance JEV infection of macrophages, further supporting this concept.

For these experiments, we used two different strains of JEV, the Nakayama strain, originally isolated in 1935 (66), and the more recent Laos strain (67). Our results demonstrate a higher infectivity and level of replication for the Laos strain in both porcine NEC and macrophages. Associated with this was also a higher level of induced innate immune responses in epithelial cells. The reason for these strain-related differences is unknown, and strain and genotype effects require further investigations.

The used *in vitro* NEC model has the advantage of representing fully differentiated ALI cultures containing mucus-producing goblet cells and functional cilia, which both represent first physical barriers of infection. In contrast to *in vivo* work, such cultures permit controlled kinetics studies of virus infection and basolateral and apical shedding, which is not possible *in vivo*. The same applies to the macrophage work in terms of virus replication and shedding and analyzing the impact of epithelial-cell-derived immunomodulatory factors. Such models may be used for any species and therefore permit analysis of species differences.

In conclusion, the present study indicates that the epithelium of the upper respiratory tract may represent the port of entry and exit for direct transmission of JEV between pigs, complementing previous *in vivo* work. We also show how this infection may lead to subsequent infection of myeloid cells known to be present in the submucosa. Our data are of relevance to understanding the mechanism of direct transmission for JEV and potentially other flaviviruses. Although the present work does not provide final proof that the respiratory epithelium represents the port of entry and exit during direct transmission events of JEV, our results represent an important basis for further *in vivo* investigations to confirm the cellular tropism of JEV and the kinetics of events during infection of pigs via the respiratory tract.

## MATERIALS AND METHODS

### Viruses.

For the present work, we employed the same JEV strains as in our previously published *in vivo* studies (8, 9, 12): JEV Laos, belonging to genotype 1 (CNS769_Laos_2009; GenBank accession number KC196115.1 [67]; kindly provided by R. Charrel, Aix-Marseille Université, France), and JEV Nakayama, genotype 3 (National Collection of Pathogenic Viruses, Salisbury, UK). JEV was propagated in Vero cells (ATCC CCL81) in G-MEM BHK-21 medium (Thermo Fisher Scientific, Basel, Switzerland) supplemented with 2% fetal bovine serum (FBS; Biowest, Nuaillé, France) and cultured at 37°C in a 5% CO_2_ atmosphere. Viral titers were determined in Vero cells using the immunoperoxidase monolayer assay (IPMA) with the anti-flavivirus E antibody 4G2 (ATCC HB-112). Titers were calculated and expressed as 50% tissue culture infective dose (TCID_50_) per milliliter.

### JEV infection of porcine nasal mucosa explants.

Nasal mucosa explants were isolated from four 3-month-old pigs at a local slaughterhouse. The nasal cavity was opened in the longitudinal axes, and nasal mucosa was removed in strips (3 by 8 cm). After the tissues were rinsed with minimum essential medium (MEM; Thermo Fisher) supplemented with antibiotics (100 U/ml of penicillin, 100 µg/ml of streptomycin, 0.25 µg/ml of amphotericin B, and 50 µg/ml of gentamicin; Sigma-Aldrich), they were placed onto sterile surgical compresses sodden with fresh MEM and kept on ice. Surgical compresses were cut in squares of 1.2 cm, placed in 12-well plates, and filled until the top of the compress surface with bronchial epithelial growth medium (BEGM [68]), supplemented with antibiotics (see above). Next, the nasal mucosa tissues were cut in 1-cm squares and placed onto the compresses in 12-well plates and refilled with medium, keeping the apical side of the tissue at the ALI. After incubation overnight at 37°C in 5% CO_2_, mucus was washed away with BEGM, and mucosa pieces were transferred to new empty 12-well plates without compresses. There tissues were challenged with JEV at 10^5^ TCID_50_s/ml in 400 µl, incubated for 1.5 h at 37°C, and washed 3 times with BEGM. In order to establish again the ALI conditions, mucosa was transferred onto new freshly prepared 12-well plates with compresses and fresh BEGM with antibiotics. After 2, 24, and 48 hpi one piece of tissue per condition and animal was processed for RNA extraction. At 72 hpi, additional tissues were embedded and cryopreserved in optimal cutting temperature compound (OCT; Leica Microsystems). Using a cryostat, nasal mucosa sections at a thickness of 4 μm were cut and then fixed with acetone-methanol (1:1).

### Generation of porcine NEC.

NEC were generated as previously described (68), with improvements. Briefly, nasal epithelial tissues were harvested from pigs at a local slaughterhouse, and tissues were transported on ice in phosphate-buffered saline (PBS) lacking Na_2_^−^ and Ca_2_^−^ (PBS^−/−^; supplemented with penicillin and streptomycin; Sigma). After washing and removal of fat and connective tissue, the epithelium was treated with 0.1% protease (mass/volume [m/v]; from Streptomyces griseus type XIV; Sigma) and desoxyribonuclease I (0.001% m/v; from bovine pancreas; Sigma) for release of epithelial cells. After 48 h the cells were harvested by gently scratching the apical surface and washed twice with PBS^−/−^. Between the washing steps, the cells were passed through a 100-μm cell strainer (Becton, Dickinson). Then 2 × 10^6^ cells were seeded in T75 tissue culture flasks coated with collagen types I and III (VitroCol; Sigma), in 25 ml of bronchial epithelial cell serum-free growth medium (BEGM; LHC basal medium supplemented; Thermo Fisher) supplemented with epidermal growth factor (EGF, 25 ng/ml; Thermo Fisher) and retinoic acid (50 nM; Sigma) and incubated at 37°C and 5% CO_2_. The medium was changed after 24 h and then every 2 to 3 days until confluence was reached. Cells were detached using trypsin (Sigma) and frozen in liquid nitrogen with 40% FBS and 10% dimethyl sulfoxide (DMSO; Sigma) in Dulbecco’s modified Eagle’s medium containing Glutamax and without phenol red (DMEM; Gibco, Thermo Fisher) at 2.5 × 10^6^ cells per tube. For ALI cultures, 12-well inserts with a pore size of 0.4 μm (Merck, Darmstadt, Germany) were coated with collagen type IV (Sigma), and 2 × 10^5^ cells per insert were seeded in 500 μl of BEGM (EGF, 25 ng/ml; Thermo Fisher) and 1 ml in the lower chamber, and cells were cultured at 37°C and 5% CO_2_. After 24 h, the insert medium was replaced, and then the medium in both chambers was changed every 2 to 3 days. After reaching confluence, the cells were shifted to ALI by removing the medium in the upper chamber, and in the basal chamber BEGM was replaced with ALI medium (1:1 [vol/vol] LHC basal medium plus DMEM, supplemented as BEGM with 1 ng/ml of EGF; Thermo Fisher). Experiments were performed as soon as movement of cilia was visible, indicating full differentiation of cultures.

### Flavivirus infection of porcine NEC.

NEC were apically challenged with an MOI of 0.1 TCID_50_/cell for 1.5 h at 37°C. Then the virus inoculum was discarded, the NEC apical surface washed three times with prewarmed PBS^−/−^, and the inserts were transferred into new plates with fresh ALI medium in the basolateral chamber for culture at 37°C and 5% CO_2._ Mock infection controls were included. After 24, 48, and 72 hpi, apical washes and basolateral medium were collected and stored at −70°C for later titrations. RNA was extracted from NEC at each indicated time postinfection. An extra insert was kept for 72 hpi to fix in 4% paraformaldehyde (PFA) for immunolabeling.

### RNA extraction and RT-qPCR.

RNA was extracted using NucleoSpin RNA II filtered columns (Macherey-Nagel). cDNA was generated with the Omniscript reverse transcription (RT) kit (Qiagen) using random hexamers (Thermo Fisher). RT-quantitative PCR (qPCR) was performed using TaqMan Fast Universal PCR master mix (Applied Biosystems). The primer and probe sequences used in this work are listed in Table 1. Fold change of gene expression was calculated using the threshold cycle (2^−ΔΔ^*^CT^*) method (69), and the 18S housekeeping gene was used for normalization. Relative gene expression results are presented on a base 2 logarithmic scale.

**TABLE 1:** Primers and probes used for qPCR

Gene	Primer or probe^*a*^	Sequence (5′–3′)^*b*^	Concentration (nM)	Reference
JEV	PF	GGTGTAAGGACTAGAGGTTAGAGG	400	74
	PR	ATTCCCAGGTGTCAATATGCTGTT	400	
	Probe	FAM-CCCGTGGAAACAACATCATGCGGC-TAMRA	100	
18S	PF	CGCCGCTAGAGGTGAAATTC	400	75
	PR	GGCAAATGCTTTCGCTCTG	400	
	Probe	FAM-TGGACCGGCGCAAGACGGA-TAMRA	100	
pIL-6	PF	CTGGCAGAAAACAACCTGAACC	400	This study
	PR	TGATTCTCATCAAGCAGGTCTCC	400	
	Probe	FAM-TGGCAGAAAAAGACGGATGC-TAMRA	100	
pIL-8	PF	CCGTGTCAACATGACTTCCAA	500	76
	PR	GCCTCACAGAGAGCTGCAGAA	500	
	Probe	FAM-TTCTTCGCCCTCAGTGTGAA-TAMRA	125	
IFN-λ 3	PF	GCCAAAGATGCCTTAGAAGAG	500	77
	PR	CAGAACCTTCAGCGTCAGG	500	
	Probe	FAM-CGCGATCGCAAGTGCCGCTCCCGCCTCTGATCGCG-TAMRA	125	
SOCS1	PF	TTCTTCGCCCTCAGTGTGAA	500	78
	PR	GGCCTGGAAGTGCACGC	500	
	Probe	FAM-TTCGGGCCCCACAAGCATCC-BHQ1	125	
CCL2	PF	CCATCAGCTCCCACACCGAA	500	This study
	PR	AAGGACCTGGGTGCAGAAGG	500	
	Probe	FAM-TGCAGCCCTCCTGTGCCTGCTGC-TAMRA	125	
CCL5	PF	TCCATGGCAGCAGTCGTCTT	500	This study
	PR	CAGGCTCAAGGCTTCCTCCA	500	
	Probe	FAM-ACCGCCAGGTGTGTGCCAACCCAGA-TAMRA	125	
CXCL10	PF	TTGAAATGATTCCTGCAAGTCAA	500	79
	PR	GACATCTTTTCTCCCCATTCTTTT	500	
	Probe	FAM-CTTGCCCACATGTTGAGATCATTGCCAC-TAMRA	125	

a PF, forward primer; PR, reverse primer.

b FAM, 6-carboxyfluorescein; TAMRA, 6-carboxytetramethylrhodamine.

### Cytokine measurements.

Basolateral supernatants of NEC harvested at 24, 48, and 72 hpi were used for determination of porcine IL-1β, IL-6, IL-8, and TNF using commercial enzyme-linked immunosorbent assay (ELISA) kits (R&D Systems, Abingdon, UK).

### Porcine macrophages preparation and infection.

MDM were generated from monocytes as previously described (70). To this end, blood from 6- to 24-month-old specific-pathogen-free (SPF) Swiss Large White pigs from our own breeding facilities was taken, and peripheral blood mononuclear cells (PBMCs) were isolated using density centrifugation (1.077 g/liter; Amersham Pharmacia Biotech). Then monocytes were isolated by magnetic cell sorting with an LS column (Miltenyi Biotec, Bergisch Gladbach, Germany) as CD172a^+^ cells using monoclonal antibody (MAb) clone 74-22-15 (ATCC). Sorted monocytes were seeded at 5 × 10^5^/ml in DMEM–10% FBS and porcine macrophage colony-stimulating factor (M-CSF; 20 U/ml, produced in-house [70, 71]) and cultured for 3 days at 39°C and 5% CO_2_. After MDM differentiation, medium was replaced with fresh MDM (for nonpolarized MDM) or with medium supplemented with IL-4 (100 U/ml, produced in-house; for IL-4-polarized MDM) (72, 73) and incubated for another 24 h.

To investigate the ability of JEV released to the basolateral side of porcine NEC to infect MDM, the cells were incubated with 200 µl of the basolateral medium collected at 72 hpi from JEV-infected NEC. As controls, MDM were challenged with JEV from our virus stocks at an MOI adapted to that of the basolateral NEC medium. To determine if epithelial alarmins influence JEV infection (Laos; MOI, 1 TCID_50_/cell) of MDM, recombinant human IL-25, IL-33, TSLP (Thermo Fisher), or any possible combination thereof was added at 10 ng/ml each, with the virus inoculum and removed at the same time after incubation. For MDM infection, the cells were incubated 1.5 h at 37°C and 5% CO_2_ with the virus, washed three times with PBS^−/−^, and incubated at 39°C and 5% CO_2_ for 24 h in fresh DMEM–2% FBS. The MDM were harvested as cell suspensions, immunolabeled with the anti-flavivirus group antigen antibody 4G2, and acquired on a FACSCantoII (Becton, Dickinson). Dead cells were excluded by electronic gating in forward/side scatter plots, followed by exclusion of doublets using Flowjo V.9.1 software (Treestar, Ashland, OR).

### Ethics statement.

All procedures which involve animals performed at the Institute of Virology and Immunology (IVI) comply with the Animal Welfare Act (TSchG SR 455), the Animal Welfare Ordinance (TSchV SR 455.1), and the Animal Experimentation Ordinance (TVV SR 455.163) of Switzerland. All studies were reviewed by the ethical committee for animal experiments of the canton of Bern, Switzerland, and approved by the cantonal veterinary authorities (Amt für Landwirtschaft und Natur, Veterinärdienst). Blood sampling was approved with license number BE88/14. Nasal mucosa was obtained in the context of regular slaughter of pigs at the slaughterhouse of the IVI. The pigs were not slaughtered for the purpose of organ collection. Consent for collecting samples after slaughter was obtained from the animal facility manager of the IVI.

### Monocyte chemotaxis assay.

First, 600 µl per well of basolateral supernatants from JEV-infected porcine NEC were harvested at 72 hpi and dispensed into 24-well plates. Next, 10^6^ monocytes were added into Transwell inserts with 3-µm-diameter pores (Becton, Dickinson) and placed in the wells filled with the porcine NEC culture-derived basolateral medium. After 3 h of incubation at 39°C and 5% CO_2_, basolateral medium was harvested and total monocytes were harvested and quantified. Monocyte phenotype was verified by immunolabeling with anti-porcine CD14 (MIL2; Bio-Rad). Controls included ALI medium, medium collected from mock-treated porcine NEC, and porcine CCL2 (200 pg/ml in ALI medium; Kingfisher Biotech, St. Paul, MN). For monocyte quantification by flow cytometry, CountBright absolute counting beads (Thermo Fisher) were used following the manufacturer’s manual. Dead cells were excluded by electronic gating in forward/side scatter plots, followed by exclusion of doublets.

### Confocal microscopy imaging.

Porcine nasal mucosa tissue slices 4 µm thick were washed in confocal buffer (CB; 50 nM ammonium chloride and 0.1% saponin in PBS^−/−^) for 30 min and then incubated with anti-flavivirus group antigen antibody 4G2 for 2 h. After 3 washes with CB, the tissue was incubated for 1 h with Alexa Fluor 488-coupled anti-IgG2a (Thermo Fisher), and Cy3-conjugated anti-β-tubulin (Abcam, Cambridge, UK) was used for the detection of cilia. Porcine NEC cultured on the Transwell membranes were washed with PBS^−/−^ and incubated in 4% PFA for 15 min. In the second step, the membranes were incubated in CB for 30 min. Cells were incubated with anti-ZO-1 (Thermo Fisher) and anti-flavivirus group antigen antibody 4G2 for 2 h, followed by Alexa Fluor 633-coupled goat anti-rabbit IgG (Thermo Fisher), Alexa Fluor 488-coupled anti-IgG2a (Thermo Fisher), and Cy3-conjugated anti-β-tubulin (Abcam). For evaluation of cell death in porcine NEC, Abcam’s Fixable Cell Viability Assay Kit (Fluorometric-Blue)-CytoPainter was applied for 30 min, and then cells were washed with PBS^−/−^ and fixed with 4% PFA, followed by an incubation with anti-flavivirus group antigen antibody 4G2 as described before and labeling with phalloidin conjugated with AF555 (Thermo Fisher) and Alexa Fluor 488-coupled anti-IgG2a (Thermo Fisher) for 1 h. As a positive control for cleaved caspase 3 (C-Cas3) staining, apoptosis in porcine NEC was induced by incubation with anti-human CD90 (5E10; Becton, Dickinson). For detection of apoptotic cells, inserts were fixed with 4% PFA and incubated in CB for 30 min, incubated with anti-C-Cas3 (Cell Signaling Technologies), anti-flavivirus E protein antibody 4G2 for 2 h, washed, and labeled with phalloidin conjugated with AF647, Alexa Fluor 633-coupled goat anti-rabbit IgG, and Alexa Fluor 488-coupled anti-IgG2a (Thermo Fisher) for 1 h. All incubations were performed at room temperature in the dark. For each staining, 4′,6-diamidino-2-phenylindole dihydrochloride (DAPI, Sigma) at 2 µg/ml was applied for 5 min at 37°C and washed off with PBS^−/−^. Then tissue slices of porcine NEC were mounted on glass slides in Mowiol 4-88 reagent (Sigma). For confocal microscopy analysis, a confocal microscope A1 (Nikon AG) combined with an ECLIPSE Ti inverted microscope (Nikon) and digital imaging Nikon software (AR 3.30.02) were used. The image acquisitions were performed with the 40× objective, sequential channel acquisition and not simultaneous was employed; in order to give high-resolution images, the acquiring setting was performed with optimized voxel size and automatic threshold. The images were analyzed with Imaris 8.0.2 software (Bitplane AG, Zurich, Switzerland). To avoid false-positive emissions, different settings were applied, including background subtraction, threshold applications, gamma correction, and maxima. Insert surface staining (for E protein, CytoPainter, and C-Cas3) was calculated as average of positive signal of each channel from 20 different nonoverlapping fields in triplicate porcine NECs using confocal microscopy.

### Statistics.

Figures and data collection analysis were done using GraphPad Prism 7 software (GraphPad Software, San Diego, CA). For viral titrations, differences between groups were assessed by Kruskal-Wallis analysis, and for individual differences the Mann-Whitney *U* test with Bonferroni correction as *post hoc* was employed. For the rest of the comparisons, such as differences between groups in the percentage of infected cells or gene expression levels, we employed a two-way analysis of variance (ANOVA). A *P* value lower than 0.05 was considered statistically significant.
